# Effects of Prior Knowledge on Decisions Made Under Perceptual vs. Categorical Uncertainty

**DOI:** 10.3389/fnins.2012.00163

**Published:** 2012-11-16

**Authors:** Kathleen A. Hansen, Sarah F. Hillenbrand, Leslie G. Ungerleider

**Affiliations:** ^1^Laboratory of Brain and Cognition, National Institute of Mental Health, National Institutes of HealthBethesda, MD, USA; ^2^Helen Wills Neuroscience Institute, University of CaliforniaBerkeley, CA, USA

**Keywords:** prior probability, expectation, frontoparietal, dorsolateral prefrontal cortex

## Abstract

Humans use prior knowledge to bias decisions made under uncertainty. In this fMRI study we predicted that different brain dynamics play a role when prior knowledge is added to decisions made under perceptual vs. categorical uncertainty. Subjects decided whether shapes belonged to Category S – smoother – or Category B – bumpier – under both uncertainty conditions, with or without prior knowledge cues. When present, the prior knowledge cue, 80/20 or 50/50, indicated that 80 and 20% (or 50 and 50%) were the chances that responding “S” and “B” (or vice versa) would be correct. During perceptual uncertainty, shapes were degraded with noise. During categorical uncertainty, shapes were ambiguous. Adding the 80/20 cue increased activation during perceptual uncertainty in bilateral lateral occipital (LO) cortex and left middle frontal gyrus (MidFG), and decreased activity in bilateral LO cortex during categorical uncertainty. Right MidFG and other frontoparietal regions were active in all conditions. The results demonstrate that left MidFG shows activation changes, suggestive of an influence on visual cortex, that depend on the factor that makes the decisions difficult. When sensory evidence is difficult to perceive, prior knowledge increases visual cortical activity. When the sensory evidence is easy to perceive but difficult to interpret, prior knowledge decreases visual cortical activity.

## Introduction

Studies of perceptual decisions made under uncertainty use various methods to define and control uncertainty. One common approach is to ask subjects to make decisions about targets degraded with noise. We call this type of uncertainty *perceptual uncertainty* because difficulty perceiving the sensory evidence in the noise is the limiting factor on accuracy. Another approach is to ask subjects to make decisions about targets that are members of overlapping categories, such that some targets are ambiguous and could belong to either category. We call this type of uncertainty *categorical uncertainty* because the sensory evidence, though easy to perceive, is difficult to interpret.

Historically, researchers testing sensory and systems neuroscience hypotheses typically choose to use perceptual uncertainty, while researchers testing neuroeconomic and cognitive neuroscience hypotheses are more likely to use conditions analogous to categorical uncertainty. In both uncertainty conditions, when prior knowledge indicates that one alternative is likelier than another, subjects bias their decisions in favor of the indicated alternative (Green and Swets, 1966). However, the neural mechanism(s) underlying this behavioral effect are not well understood.

In this study, we show that modulatory effects obtained in the laboratory using perceptual uncertainty may not generalize to conditions of categorical uncertainty, and vice versa. These results may be valuable to researchers seeking to interpret data and design translational studies bridging different subfields of neuropsychology. For example, in ecological contexts, the ability to apply prior knowledge during conditions of perceptual uncertainty may be highly adaptive. If an organism knows that there are tigers in the region, it makes sense for that organism to “see” a barely perceptible shape in the shadows as a likely tiger. In contrast, there are other contexts – for example, financial – in which categorical, not perceptual, uncertainty is the bottleneck making decisions difficult. When a person makes decisions about how to interpret a number representing a price, the decision process generally does not depend on the legibility of the digits. More commonly, the digits are clearly perceived, but may be difficult to categorize as too high or too low.

In previous fMRI studies (Hansen et al., 2011, 2012), we manipulated prior knowledge during decisions about visual stimuli in categorical uncertainty only. Instead of asking subjects to make decisions about abstract items such as numbers, we asked them to categorize shapes that differed along the single, quantitative dimension of curvature. These studies showed that prior knowledge altered fMRI activity levels in prefrontal and parietal cortex, but did not reveal enhanced activity from prior knowledge in visual cortex. The absence of an effect in visual cortex was surprising, because it seemed to be at odds with published observations documenting that cues providing subjects with expectations about visual stimuli enhanced activity in stimulus-selective visual cortex (Eger et al., 2007; Summerfield and Koechlin, 2008; Esterman and Yantis, 2010).

We wondered whether this lack of an effect in visual cortex might be due to the fact that decisions in our studies were made under categorical uncertainty only. Our reasoning here was that given sensory stimuli that were ambiguous but not degraded with noise, substantial internal modulation of the sensory evidence would amount to misperception. In contrast, during perceptual uncertainty, when sensory stimuli are noisy, it could be adaptive for prior knowledge to enhance the representation of the evidence itself. Therefore, we hypothesized that prior knowledge would increase activity in sensory processing regions in decisions made under perceptual uncertainty but not categorical uncertainty.

To test this hypothesis, we asked subjects to categorize curved shapes under perceptual uncertainty, with and without prior knowledge, and compared the resulting behavioral and fMRI data with the previously published categorical uncertainty data. Differences in activation between the perceptual and the categorical uncertainty conditions were identified in left middle frontal gyrus (MidFG) and in bilateral lateral occipital (LO) cortex. In all three regions, activation levels were greater during perceptual than categorical uncertainty. Breaking down the within-regions of interest (ROI) data into prior knowledge and naïve subject groups revealed that the activation differences observed in the pooled data were driven by the prior knowledge group. In the perceptual uncertainty condition, activations in left MidFG and bilateral LO were higher for prior knowledge subjects than naïve subjects, while in the categorical uncertainty condition, activations in left MidFG and bilateral LO were lower for prior knowledge subjects than naïve subjects. The sign of the activations was positive in all conditions in the occipital regions. In left MidFG, the activation was positive during perceptual uncertainty with prior knowledge and negative in the other conditions. Right MidFG and other regions previously implicated in executive control and decisions were positively activated in all conditions, but the activation levels did not differ across uncertainty type. Thus, positive MidFG activation was right-biased in three of the four experimental conditions, and activation was seen in both right and left MidFG only during perceptual uncertainty with prior knowledge.

These findings indicate that left MidFG shows activation changes, suggestive of an influence on visual cortex, that depend on the factor that makes the decisions difficult. Given prior knowledge when the limiting factor is perceptibility, right prefrontal activity is accompanied by positive activity in left prefrontal cortex and enhanced positive activity in sensory processing regions. In contrast, when the sensory evidence is easy to perceive but difficult to interpret, prior knowledge results in right-biased prefrontal activity accompanied by decreased positive activity in sensory processing regions.

## Materials and Methods

### Participants

In this study, we report fMRI and behavioral data from 66 subjects (34 male) of mean age 25 years (range 20–41). All subjects provided informed consent before the experiment. All procedures were approved by the National Institute of Mental Health Institutional Review Board. All subjects were right-handed and had normal or corrected-to-normal vision. Of the subjects, 22 made decisions under perceptual uncertainty with prior knowledge; 22 made decisions under categorical uncertainty with prior knowledge; and 22 made decisions under both uncertainty conditions with no prior knowledge. For the first subject group, we acquired data from 26 subjects but excluded data from four because *d*′ from the scanning data was more than 2 SDs below the mean of the other subjects’ *d*′ (two subjects) or a decision criterion shift was observed in the non-predicted direction (two subjects). The last two subject groups were described in a previous publication in which we used a different approach to analyze the datasets (Hansen et al., 2011); the perceptual uncertainty data are presented here for the first time.

### Stimuli and task

**Figure 1:**
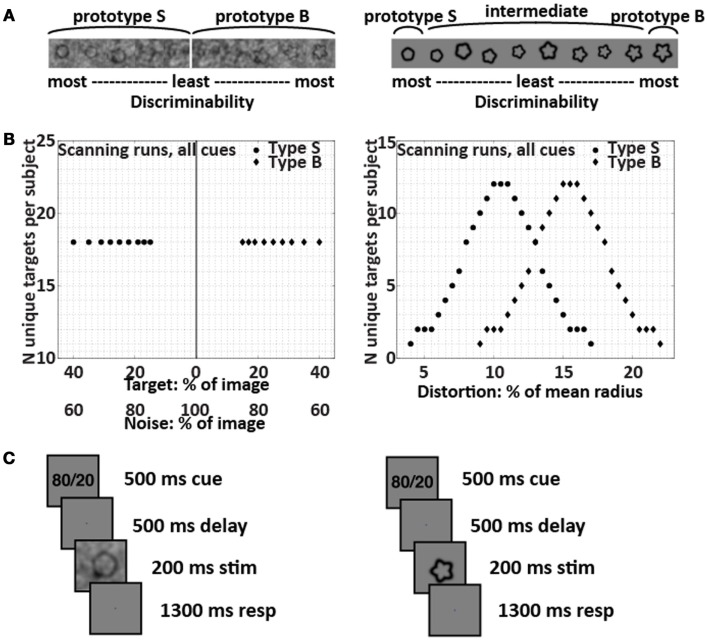
**Targets and trial structure**. The SNR-modulated targets that created perceptual uncertainty are shown on the left, and the form-modulated targets that created categorical uncertainty are shown on the right. **(A)** Example targets. The SNR-modulated targets were either extremely smooth or extremely bumpy and were overlaid with varying amounts of noise; each individual noise pattern was unique. The form-modulated targets were of varying degrees of curvature and were not overlaid with noise. **(B)** Target distributions used in all scanning runs for all subjects. **(C)** Trial structure in scanning runs. The subjects’ task was to decide whether each target belonged to Category S or B. Depending on subject group and run, the cue was 80/20, 50/50, or OO/OO. The set of images used to provide the 200-ms stimuli for the trials were identical across the 80/20, 50/50, and OO/OO scanning runs; only the cue changed.

Subjects used two fingers of the right hand to press buttons to report decisions about visual targets. In the categorical uncertainty condition, targets were form-modulated. The form-modulated targets were distorted circles with sinusoidal modulation ranging linearly from 4 to 22% of the mean radius, with a step size of 0.5% (Figure 1). No noise obscured the form-modulated targets. Distributions of Category S and B form-modulated targets were Gaussian and overlapping (Healy and Kubovy, 1981; Maddox, 2002). The overlapping distributions (Figure 1) made the intermediate form-modulated targets ambiguous, so that the targets alone would not contain sufficient information for subjects to classify them with perfect accuracy. In the perceptual uncertainty condition, targets were signal-to-noise-modulated (SNR-modulated). The SNR-modulated targets were distorted circles (Wilkinson et al., 1998) with sinusoidal modulation of either 4% (Category S for smooth) or 22% (Category B for bumpy) of the mean radius, obscured with noise (Figure 1). The noise pattern used in each image was unique. We created the noise patterns by combining an original target’s power spectrum with random phases and converting this information back to image space via inverse Fourier transform. Each target was overlaid with its own noise pattern, using one of nine different weight ratios that ranged from 15% target + 85% noise to 40% target + 60% noise. The weights were derived from pilot studies done outside the scanner in order to equate behavioral performance (as measured by *d*′ and by the magnitude of the criterion shift between the 80/20 and 50/50 prior knowledge conditions) during perceptual uncertainty relative to categorical uncertainty. In all, cases, targets were presented one at a time with random sizes, orientations, and locations to prevent subjects from relying on retinotopic location or spatial attention in order to perform well.

Before entering the scanner, all subjects underwent behavioral training that provided instant feedback after each trial. For the subjects making decisions with prior knowledge, the explicit prior knowledge cue “80/20” in some runs and “50/50” in other runs preceded each SNR-modulated or form-modulated shape. The indicated target category – that is, the category indicated by 80 in the 80/20 runs – was either S or B for each subject. The 80/20 training runs were comprised of 80% indicated and 20% contraindicated targets, and the 50/50 training runs were comprised of 50% indicated and 50% contraindicated targets. Thus, during training, the explicit prior knowledge cues reflected the implicit prior probability distributions of the targets. The training distributions were created by manipulating the prior probability of occurrence of the physical targets themselves, rather than changing the category boundary. The prior knowledge subjects were informed explicitly that the target distributions were either 80% indicated and 20% contraindicated or 50% of each, and their understanding of this concept and the task was confirmed by their answers to questions during pre-training instruction.

For the naïve subjects, a sham cue “OO/OO” preceded each SNR-modulated or form-modulated shape. The subjects were told that they could think of the letter O’s as open eyes reminding them to keep looking at the screen. Except for the cue, the training runs for the naïve subjects were identical in all respects, including the target images, to the 50/50 training runs for the prior knowledge subjects. Thus, the training runs for the naïve subjects were comprised of 50% S and 50% B targets, although the subjects were not informed explicitly of this fact. In fact, except for the cue, all aspects of the training runs for the naïve subjects, including the target image sets, were identical to the training runs used for the prior knowledge subjects at 50/50. The subjects’ understanding of the task was confirmed by their answers to questions during pre-training instruction.

During scanning, no subject received feedback. In one-third of the scanning trials, a blank screen took the place of the target and subjects were instructed to make no response; including these blank trials permitted us to obtain estimates of activity during decision vs. blank trials. The only difference in the runs for the prior knowledge subjects vs. the naïve subjects was that the cues – as in the training runs – were 80/20 or 50/50 for the prior knowledge subjects and OO/OO for the naïve subjects. Importantly, for all subjects, all scanning runs were comprised of 50% S and 50% B targets, and the target images themselves were identical in every respect for all subjects. This control ensured that differences between prior knowledge conditions could be attributed only to the cue and not to stimulation differences.

The order of trial types (Category S target, Category B target, or blank) for the scanning runs was determined by assigning each run a different ternary m-sequence. m-Sequences are efficient in terms of signal per time, especially for relatively short scan durations, and are exactly counterbalanced over time, minimizing any uncontrolled adaptation or expectation effects (Sutter, 2001; Buračas and Boynton, 2002). m-Sequences were generated using code written by Buračas and Boynton (2002). Each run-length m-sequence was length 3^4^ − 1 = 80 trials, consisting of 27 Category S stimulus trials, 27 Category B stimulus trials, and 26 blank trials. Each trial lasted 2.5 s. A blank grayscale screen was shown for 10 s at the beginning of each run to allow the magnetic field to reach equilibrium and for 12.5 s at the end of each run to allow for the delay in the hemodynamic response. The data presented here represent six runs at 80/20 and six runs at 50/50 from each prior knowledge subject, under either perceptual or categorical uncertainty, and six runs under perceptual and six runs under categorical uncertainty from each naïve subject.

### Imaging data acquisition and preprocessing

All MRI data were collected on a GE 3-T scanner with a GE whole-head eight-channel coil. For fMRI we used an EPI (echo-planar imaging) sequence with TR (repetition time) = 2.5 s per shot (=2.5 s per acquired brain volume), TE (echo time) = 30 ms, field of view 22 cm × 22 cm, resolution 64 × 64 voxels per slice (in-plane voxel size 3.4 mm × 3.4 mm), and slice thickness 3.0 mm. Each fMRI brain volume consisted of 38 axial slices. For anatomical images we used an MP-RAGE (magnetization prepared rapid acquisition gradient echo) sequence with field of view 24 cm × 24 cm, 128 locations per slab, and slice thickness 1.2 mm. Unless otherwise noted, preprocessing and subsequent analysis of the MRI data was performed with the AFNI software package (Cox, 1996; Cox and Hyde, 1997). The first four brain volumes of every fMRI run were removed; brain volumes were shifted to account for slice acquisition time and motion-corrected. Each subject’s T1-weighted anatomical dataset was warped via 12-parameter affine transform to the TT-N27 brain template.

### ROI identification

To identify ROIs as a test of our main hypothesis – that prior knowledge would increase activity in sensory processing regions in decisions made under perceptual uncertainty but not categorical uncertainty – we used a general linear model (GLM) in which the regressor of interest was a sequence of 0’s and 1’s convolved with a model hemodynamic function. The 0’s and 1’s represented blank and decision trials respectively. The outputs of each GLM were voxelwise beta weights representing decision trial activity for a single subject in one condition, where the six possible conditions were perceptual uncertainty with prior knowledge at 80/20, perceptual uncertainty with prior knowledge at 50/50, perceptual uncertainty without prior knowledge, categorical uncertainty with prior knowledge at 80/20, categorical uncertainty with prior knowledge at 50/50, and categorical uncertainty without prior knowledge. Using a two-tailed *t*-test on data from the 80/20 and naïve conditions, pooled across all subjects, we calculated the group voxelwise significance of the absolute value of the difference between the beta weights from the perceptual uncertainty vs. categorical uncertainty conditions. ROIs were located by limiting surviving clusters in the group results to regions with *p*-values < 0.05, corrected for multiple comparisons across voxels. Cluster coordinates were determined by affine registration to the TT-N27 brain template.

In a subsequent test we located regions with positive decision-related fMRI activity in the conjunction of four conditions: 80/20 perceptual uncertainty, naïve perceptual uncertainty, 80/20 categorical uncertainty, and naïve categorical uncertainty. Using a two-tailed *t*-test pooled across all subjects, we calculated the group voxelwise significance of the mean activation level for each condition. ROIs were located by limiting surviving clusters to regions with *p*-values < 0.05, corrected for multiple comparisons across voxels and experimental conditions. The conjunction here was the strict conjunction of conditions. As in Nichols et al. (2005), we used a test for a logical AND by requiring that all the comparisons in the conjunction were individually significant: to obtain the corrected *p* < 0.05 across conditions, we required positive activations of *p* < 0.0125 in every one of the four conditions. Cluster coordinates were determined by affine registration to the TT-N27 brain template.

We also located regions where activity, as defined by linear covariation with the degree of uncertainty, differed across uncertainty conditions. The object here was to test for the possibility that although no regions exhibited greater average activity during categorical than perceptual uncertainty, some regions’ activity covaried with categorical but not perceptual uncertainty and vice versa. We performed ROI searches using this approach on the naïve and 80/20 data independently. In each prior condition, we performed a whole-brain search and a search constrained to perirhinal cortex and anterior temporal lobe, which are known to be responsive for learned visual categories. With the exception of the regressors used in the GLM analyses of individual subject data, these analyses were essentially equivalent to that defining the ROIs for our main hypothesis (above). In the covariation analysis, the regressor of interest was a sequence of numbers ranging between 0 and 1, convolved with a model hemodynamic function. Before convolution, target trials were represented by a number between 0 and 1 equivalent to the distance from the target distribution’s nearest extreme to its midpoint. Thus, midpoint targets received a value of 1 (representing complete uncertainty), endpoint targets received a value of 0 (representing no uncertainty), and intermediate targets received values scaling proportionately. Blank trials received values of 0.

## Results

### Behavior

**Figure 2:**
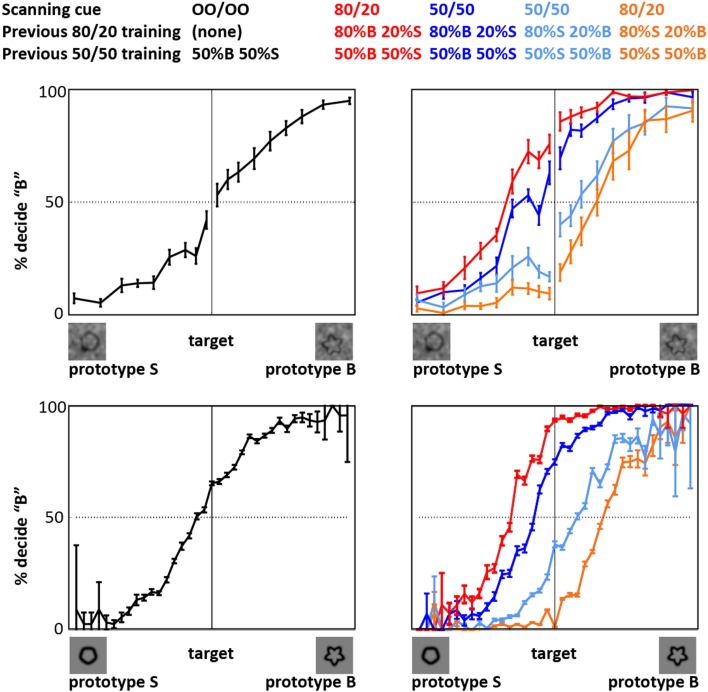
**Decision behavior during fMRI scanning**. The plots show that priors training biased decision reports, relative to naïve subjects, in both perceptual uncertainty (top) and categorical uncertainty (bottom). Black: naïve subjects. Red: prior knowledge subjects whose pre-scan training indicated that the likelier category to appear was Category B (bumpier); behavior given the 80/20 cue. Relative to the other subjects, the decision reports of these subjects are biased in favor of a “B” response. Dark blue: behavior of the same subjects given the 50/50 cue. Orange: prior knowledge subjects whose pre-scan training indicated that the likelier category to appear was Category S (smoother); behavior given the 80/20 cue. Relative to the other subjects, the decision reports of these subjects are biased in favor of an “S” response. Light blue: behavior of the same subjects given the 50/50 cue. Error bars represent ±1 SE across subjects. In the categorical uncertainty plots (bottom), the error bars tend to be very small at intermediate and larger at extreme stimulus levels. This pattern reflects the fact that the form-modulated target distribution included many intermediate, i.e., ambiguous, targets, and relatively few extreme, i.e., unambiguous, targets.

The behavioral data acquired during fMRI data acquisition (Figure 2) indicated that training with the prior knowledge cues induced a decision bias during the fMRI experiment. In this paper, the term *prior knowledge subjects* refers to subjects trained in conditions that both implicitly and explicitly indicated that one of two target categories was likelier to be presented than the other. We refer to prior knowledge subjects trained that Category S (or B) was the likely category as Group S (or B) prior knowledge subjects. The term *naïve subjects* refers to subjects trained in conditions that did not implicitly or explicitly indicate either category as more likely than the other. For details about the training, see Section “Materials and Methods” and Figure 1. During the fMRI experiment, Group S prior knowledge subjects working under both perceptual and categorical uncertainty responded “S” (or “B”) for a given shape more often than did the naïve subjects making decisions about the same shapes (Figure 2). That is – unsurprisingly – prior knowledge about the stimuli biased subjects’ decisions in favor of the expected stimulus type. Under both uncertainty conditions, given the 50/50 prior knowledge cue, the prior knowledge subjects retained a persistent, though diminished, bias of the same sign as their bias in the 80/20 condition; for an in-depth examination of this phenomenon in the categorical uncertainty condition, see Hansen et al. (2011).

**Figure 3:**
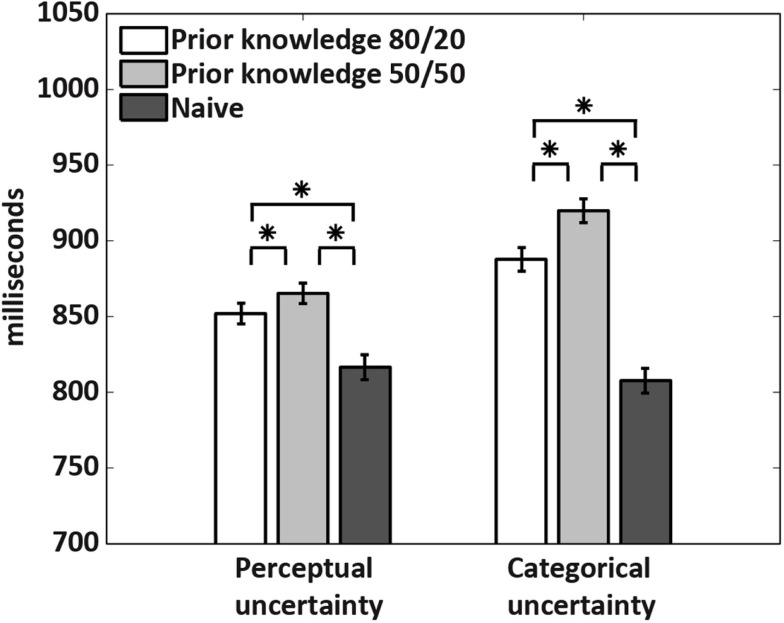
**Response times**. The 80/20 cue gave a speed advantage to the prior knowledge subjects relative to the 50/50 cue in the same subjects. Across the prior knowledge subjects, response times were longer for the subjects performing under categorical than perceptual uncertainty. This difference was not seen in the naïve subjects. Stars indicate *p* < 0.0001, calculated via a two-tailed *t*-test across conditions.

To obtain a first indication of the mechanisms underlying the decision bias induced by prior knowledge, we examined response times (RTs) in all subjects (Figure 3). In the prior knowledge subjects, RTs were shorter at 80/20 than 50/50 in both uncertainty conditions, demonstrating that prior knowledge about the stimuli conferred a speed advantage regardless of uncertainty type. In the prior knowledge subjects, RTs were also shorter for subjects performing under perceptual than categorical uncertainty. This observation suggests that the mechanism by which prior knowledge is integrated into decisions differs when the decisions are made under perceptual vs. categorical uncertainty. Importantly, RTs in the naïve subjects did not differ during perceptual vs. categorical uncertainty, implying that our effort to match difficulty across uncertainty types by adjusting the noise weights in the perceptual uncertainty condition was successful.

### Imaging data

**Figure 4:**
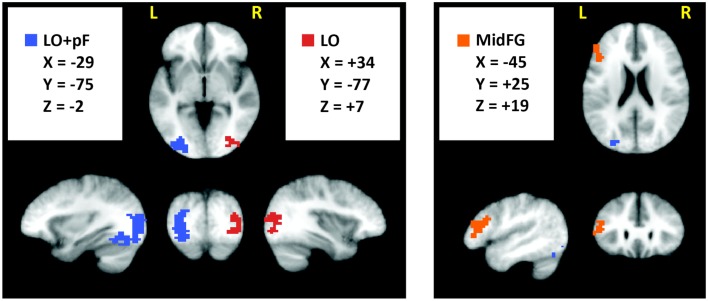
**Brain regions selective for uncertainty condition**. We pooled the 80/20 and naive fMRI datasets across all subjects and searched for regions with a significant (*p* < 0.05, corrected) difference, of either sign, between the perceptual and categorical uncertainty conditions. Surviving clusters are shown here, overlaid on an average of the anatomical images from all subjects.

The current study was designed to reveal differences in how the brain integrates prior knowledge into decisions during perceptual vs. categorical uncertainty. To investigate this topic, we first identified ROIs in which activation levels were different in decision trials made under perceptual vs. categorical uncertainty, pooled across all subjects (Materials and Methods). The ROI locations – left MidFG, left LO and posterior fusiform (LOpF) cortex, and right LO are shown in Figure 4, and their coordinates and volumes are listed in Table 1. These results show that left MidFG, left LO/pF, and right LO responded differentially to the perceptual uncertainty and the categorical uncertainty conditions.

**Table 1:** **Brain regions selective for uncertainty condition**.

Location	*x*	*y*	*z*	Volume (mm^3^)
Middle frontal gyrus (MidFG)	−44.6	25.4	18.6	3042
Lateral occipital cortex and posterior fusiform cortex (LO + pF)	−29.4	−74.7	−1.8	9822
Lateral occipital cortex (LO)	34.4	−77.2	6.6	4406

*This table provides the coordinates and volumes of voxel clusters responding with activation differences (*p* < 0.05, corrected) between decision trials under perceptual vs. categorical uncertainty, pooled across subjects. Negative (positive) values of *x* indicate the left (right) hemisphere*.

**Figure 5:**
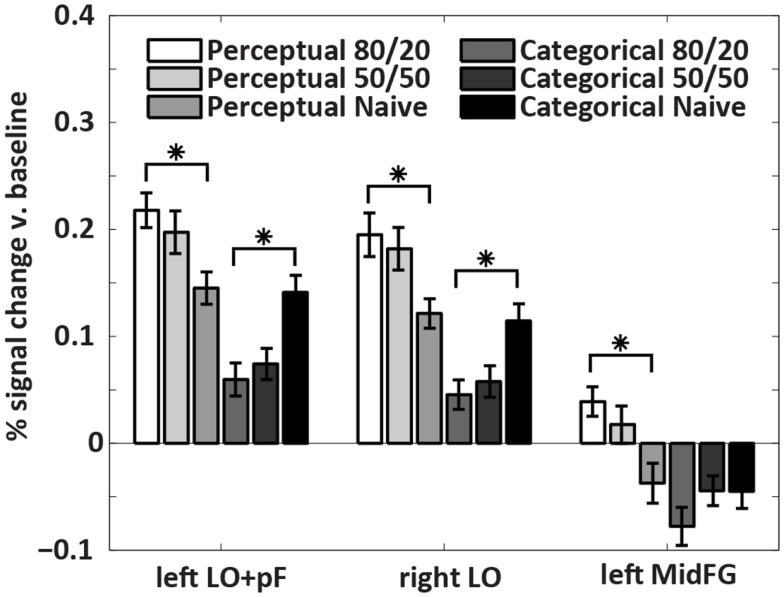
**Within-ROI results**. The bar charts show mean brain activity across all decision trials, relative to blanks, in each condition. Stars indicate differences of *p* < 0.05 as calculated with a two-tailed *t*-test. In the naïve subjects, decisions under perceptual uncertainty evoked the same level of within-ROI activity as decisions under categorical uncertainty. Adding the prior knowledge cue to decisions under perceptual uncertainty visual targets increased activation in visual association and prefrontal cortices relative to no prior knowledge cue. Adding the prior knowledge cue to decisions under categorical uncertainty decreased activation in visual association cortex relative to no prior knowledge cue. In left MidFG, only decisions made under perceptual uncertainty with the 80/20 prior knowledge cue resulted in positive brain activations; the mean activation with the 50/50 cue was not significantly above zero.

The analysis that located the ROIs pooled the data across all subjects. To indicate whether the differences were driven by the prior knowledge subjects, the naïve subjects, or both groups, we plotted activations for each condition separately in a within-ROI bar chart (Figure 5). The chart shows that the differences were driven by the prior knowledge data. In all three ROIs, the perceptual uncertainty condition evoked the same activity level as the categorical uncertainty condition in the naïve subjects. At 80/20, the prior knowledge subjects showed greater activation than the naïve subjects in visual association (LO/pF) and prefrontal (MidFG) cortices during perceptual uncertainty. In contrast, during categorical uncertainty, at 80/20 the prior knowledge subjects showed less activation than the naïve subjects in bilateral LO (in the MidFG ROI, a trend in the same direction did not reach significance). Thus, the results supported our main hypothesis: prior knowledge increased activity in sensory processing regions in decisions made under perceptual uncertainty but not categorical uncertainty. The results also indicated a prefrontal mechanism for this effect, namely, positive activity levels in left MidFG, which occurred only during decisions made in the combination of perceptual uncertainty and prior knowledge.

The procedure for locating ROIs was based on a contrast: the absolute value of the difference between activity levels during perceptual vs. categorical uncertainty. We also wished to document the regions in which decisions in each of four conditions – perceptual uncertainty with 80/20 prior knowledge, perceptual uncertainty without prior knowledge, categorical uncertainty with 80/20 prior knowledge, and categorical uncertainty without prior knowledge – elicited positive levels of activation. Table 2 lists the coordinates and volumes of brain regions active in the strict conjunction (defined by logical AND) of the four conditions (*p* < 0.05, corrected for multiple comparisons between voxels and experimental conditions): right MidFG; left putamen (Put); two clusters in the left anterior insula (AntIns1 and AntIns2); a left hemisphere thalamic cluster (Thal) whose coordinates included those of the medial dorsal, ventral posterior medial, ventral posterior lateral, and ventral lateral nuclei; a left hemisphere cluster including postcentral gyrus, inferior parietal lobule (IPL), and intraparietal sulcus (PcG/IPL/IPS); right IPL; and large bilateral clusters covering much of ventrotemporal cortex plus some cerebellum (VT/cereb).

**Table 2:** **Brain regions active in all conditions**.

Location	*x*	*y*	*z*	Volume (mm^3^)
Middle frontal gyrus (MidFG)	44.5	33.2	22.5	533
Anterior insula, focus 1	−28.6	14.9	10.7	533
Putamen (Put)	−21.7	1.2	5.9	2769
Medial frontal gyrus (MedFG)	−2.8	0.2	49.2	4651
Anterior insula, focus 2	−38.2	−5.4	14.9	604
Thalamus (Thal): medial dorsal, ventral posterior medial, ventral posterior lateral, and ventral lateral nuclei	−12.6	−17.3	8.1	2521
Postcentral gyrus, inferior parietal lobule, and intraparietal sulcus (PcG/IPL/IPS)	−40.5	−29.8	48.7	15372
Inferior parietal lobule (IPL)	45.6	−36.4	38.5	391
Ventrotemporal cortex and cerebellum (VT/cereb)	27.7	−58.0	−16.1	20874
Ventrotemporal cortex and cerebellum (VT/cereb)	−33.7	−66.5	−14.1	9443

*This table provides the coordinates and volumes of voxel clusters responding with positive activations during decision trials in all four conditions (*p* < 0.05, corrected), where the conditions were perceptual uncertainty with prior knowledge, perceptual uncertainty without prior knowledge, categorical uncertainty with prior knowledge, and categorical uncertainty without prior knowledge. Negative (positive) values of *x* indicate the left (right) hemisphere*.

One particularly interesting observation emerging from this table is of a right hemisphere MidFG (a.k.a. dorsolateral prefrontal, DLPFC) cluster, active in all four conditions. The right MidFG cluster also overlaps with a region in which activity modulations across prior knowledge conditions were previously shown to correlate with the main effect of prior knowledge on decision behavior, i.e., a shift in the decision criterion (Hansen et al., 2012). The right MidFG cluster observed in the current study is also located at coordinates that are essentially the mirror image of the left hemisphere MidFG coordinates. Recall that the left hemisphere MidFG ROI was activated by decisions during both prior knowledge and perceptual uncertainty, but deactivated in all other conditions. Thus, across all conditions the overall pattern of activity in DLPFC was generally right-biased, becoming bilateral only when prior knowledge was combined with perceptual uncertainty.

We also located regions where activity, as defined by linear covariation with the degree of uncertainty, differed across uncertainty conditions. The object here was to test for the possibility that although no regions exhibited greater average activity during categorical than perceptual uncertainty, some regions’ activity covaried with categorical but not perceptual uncertainty and vice versa. We performed ROI searches using this approach on the naïve and 80/20 data independently. In each prior condition, we performed a whole-brain search and a search constrained to perirhinal cortex and anterior temporal lobe, which are known to be responsive for learned visual categories.

Whole-brain searches in naïve subjects revealed ROIs in left and right LO (Table 3). These overlapped with the LO ROIs identified in the main test. Activity levels in both ROIs covaried with the degree of uncertainty more during perceptual than categorical uncertainty. In prior knowledge subjects at 80/20, smaller overlapping ROIs showed the same difference sign (perceptual over categorical). An additional ROI was identified in lingual gyrus in the 80/20 data only, and in this ROI activity levels covaried more during categorical than perceptual uncertainty. However, some care should be exercised in interpreting this result, since the location of the ROI appears to be consistent with a part of early visual cortex (V1 or V2) representing far-peripheral visual space that would not have been stimulated by our targets. While we find it difficult to provide a simple explanation, we note that similar regions often appear in lists of activated regions in cognitive neuroscience papers (though the correspondence to far-peripheral V1/V2 is rarely mentioned). Possibly, some spatial attentional effect may be involved.

**Table 3:** **Brain regions selective for uncertainty condition when activity was defined as covariation with degree of uncertainty**.

Location	*x*	*y*	*z*	Volume (mm^3^)	Uncertainty preference
**NAÏVE**					
Lateral occipital cortex (LO)	−26.6	−73.4	−4.6	9042	Perceptual
	30.1	−69.3	2.8	11170	Perceptual
**80/20**					
Lateral occipital cortex (LO)	−27.1	−78.7	5.3	4680	Perceptual
	34.4	−76.3	9.2	2837	Perceptual
Lingual gyrus	−1.8	−74.5	1.7	3652	Categorical

*This table provides the coordinates and volumes of voxel clusters responding with covariation differences (*p* < 0.05, corrected) between perceptual vs. categorical uncertainty. Negative (positive) values of *x* indicate the left (right) hemisphere*.

A similar search, constrained to perirhinal cortex and anterior temporal lobe, identified one ROI (Table 4) in which activity levels covaried more during categorical than perceptual uncertainty in naïve subjects. No ROIs in this anatomical search space were identified with greater covariation for perceptual than categorical uncertainty in naïve subjects, and no ROIs in this anatomical search space were identified at all in the 80/20 data.

**Table 4:** **Brain regions selective for uncertainty condition when activity was defined as covariation with degree of uncertainty and the search space was constrained to anterior temporal lobe and perirhinal cortex**.

Location	*x*	*y*	*z*	Volume (mm^3^)	Uncertainty preference
**NAÏVE**					
Parahippocampal gyrus/BA 36	22.9	−29.3	−15.5	106	Categorical
**80/20**					
(No ROIs located at 80/20)					

*This table provides the coordinates and volumes of voxel clusters responding with covariation differences (*p* < 0.05, corrected) between perceptual vs. categorical uncertainty. Negative (positive) values of *x* indicate the left (right) hemisphere*.

One potential concern with the above observations is that the contrast used to define the key ROIs was based on a subject pool of which two-thirds were prior knowledge subjects. The potential pitfall here is a scenario in which the naïve subjects might have had ROIs in other locations, in which existing differences between the two uncertainty conditions failed to reach significance in the pooled subject dataset. To check against this possibility, we performed a separate analysis in an attempt to locate ROIs for the perceptual vs. categorical uncertainty conditions in the 22 naïve subjects only. No clusters were found that survived our statistical threshold.

## Discussion

In this study, we asked subjects undergoing fMRI scanning to make decisions about visual targets under conditions of perceptual and categorical uncertainty, with and without prior knowledge of the response that was likely to be correct. Subjects trained to use a prior knowledge cue showed larger positive activations in bilateral LO and left pF cortex during decisions made under perceptual uncertainty than did naïve subjects. Under categorical uncertainty, the prior knowledge subjects experienced smaller decision-related positive activations than did naïve subjects. In the left MidFG, the condition associated with the highest activation levels in the occipital ROIs – namely, prior knowledge during perceptual uncertainty – was the only one eliciting positive decision-related activity. During perceptual uncertainty when no prior knowledge was available and during categorical uncertainty, regardless of prior knowledge, decisions negatively activated left MidFG.

These observations enhance our understanding of the integration of prior knowledge into decision-making in several respects. First, the results demonstrate that top-down prior knowledge effects in the brain during perceptual decisions depend on the reason the decisions are difficult. Namely, the sign of the priors-related modulation in visual cortex was positive when the sensory evidence was difficult to perceive and negative when the evidence was easy to perceive but difficult to interpret. The sign difference observed across these two conditions implies that the effects of prior knowledge on perceptual decisions are not uniform across decision types, but rather depend on the attributes of the stimuli about which the decisions are made.

Besides simply establishing a dependence on stimulus attributes, the observations point to underlying neural mechanisms in each uncertainty condition. During perceptual uncertainty, decisions in the context of prior knowledge positively activated left MidFG (Figure 4; Table 1). The prefrontal decision-related activation in the prior knowledge and perceptual uncertainty condition was actually bilateral, since this condition as well as the others positively activated right MidFG (Table 2). Our observations may be related to the observation by Rahnev et al. (2011) of larger activity in the lateral prefrontal cortex, not far away from the current site of activity, when participants had prior knowledge about a perceptual decision. The bilateral MidFG activation was also associated with increased positive activation in bilateral LO and left pF cortex, visual regions selective for shapes and objects (Malach et al., 1995; Grill-Spector et al., 1998; Kourtzi and Kanwisher, 2000). This increase in activation confirmed the prediction that motivated the current study: when stimuli were noisy, such that enhancing the representation of the sensory evidence could be adaptive, prior knowledge increased activation in the relevant parts of visual cortex. The anatomical locations of the occipital ROIs are consistent with previously documented loci for shape selectivity (Malach et al., 1995; Grill-Spector et al., 1998; Kourtzi and Kanwisher, 2000), so these were precisely the regions in which signal modulation had the most potential to affect performance in our shape decision task. Thus, the perceptual uncertainty observations are analogous to a previous demonstration that prior knowledge favoring faces (or houses) enhanced fMRI activity in FFA (or PPA; Esterman and Yantis, 2010). In parallel, during categorical uncertainty, subjects with prior knowledge experienced significantly lower activation levels in the visual ROIs than did naïve subjects. The prediction motivating this study was that prior knowledge would increase visual cortical activity during perceptual but not categorical uncertainty; we did not explicitly predict that prior knowledge would actually decrease visual cortical activity during categorical uncertainty. However, the decrease is intuitive; it suggests that the prior knowledge subjects were giving less weight to the sensory evidence of curvature than were the naïve subjects. Such a strategy would be reasonable, as the RT data imply that adding prior knowledge to the categorical task imposed an additional cognitive load relative to the naïve condition. Giving less weight to visual appearance, relative to the naïve condition, may have partly compensated for an increased cognitive load.

Our observations may be relevant to those from previous studies that identified dissociations between abstract rule- or category-selective activity in prefrontal cortex and stimulus-selective activity in more posterior brain regions. For example, Jiang et al. (2007) asked subjects undergoing fMRI to make decisions about morphed cars and showed that changing perceptual vs. categorical qualities of the stimuli modulated activity in LO and right prefrontal cortex respectively. Similarly, Montojo and Courtney (2008) used a mental arithmetic task with fMRI and showed that rule updating preferentially activates prefrontal cortex while number updating preferentially activates parietal cortex.

During perceptual uncertainty without prior knowledge, and during categorical uncertainty regardless of prior knowledge, decisions negatively activated left MidFG (Figure 4; Table 1). The term negative activation, also known as deactivation, means that the fMRI signal level was lower during trials when a target was present and a decision was made than during blank trials when no target was present and no decision was made. Negative activations are seen in brain regions whose function is not relevant to the experimental condition being tested. For example, when task-relevant stimuli are visual, stimulus presentation often results in negative activation of auditory cortex (Haxby et al., 1994; Amedi et al., 2005). Concurrent negative and positive activations can also occur in left hemisphere and right hemisphere counterparts of the same cortical area. For example, stimulation of the right median nerve, which elicits positive activation in left primary somatosensory cortex, also elicits negative activation in right primary somatosensory cortex (Hlushchuk and Hari, 2006; Kastrup et al., 2008). One interpretation of such observations is that negative activations reflect suppression of functional activity that is not required for the task at hand. According to this line of reasoning, our results imply that left MidFG plays a role in integrating prior knowledge during perceptual uncertainty, but is not required during decisions in general. This conclusion is consistent with our previous results that implicated only right MidFG involvement in prior knowledge during categorical uncertainty (Hansen et al., 2011, 2012).

Our results also show that the modulation of sensory activity cannot be attributed to a general arousal effect, but rather is targeted to the part of visual cortex where a modulation could have the most impact on task performance. This can be seen by examining the location of the occipital ROIs: bilateral LO and left pF, regions already known to be selective for shapes and objects like our shape targets (Malach et al., 1995; Grill-Spector et al., 1998; Kourtzi and Kanwisher, 2000). For comparison, we did not see any effects in earlier visual areas, such as V1, V2, or V3, which are selective for the spatial location but not for the shape of visual stimuli. Since our stimuli were jittered in size, orientation, and spatial position, modulatory effects in the earlier visual areas would not be predicted to affect performance. Changes in arousal or attention have been shown to modulate signals in these earlier visual areas (Tootell et al., 1998; Watanabe et al., 1998; Somers et al., 1999; Huk and Heeger, 2000). Since no such modulation was observed in the earlier areas, we conclude that the modulation that we did observe in LO and pF was not due to overall arousal or attentional state.

During both uncertainty conditions, the decision response curves (Figure 2) and the within-ROI fMRI activity levels (Figure 5) seen in prior knowledge subjects at 50/50 tended to fall between activity levels seen in the same subjects at 80/20 and activity levels in the naïve subjects. A previous publication (Hansen et al., 2011) focuses on this interesting *persistent bias* pattern in the categorical uncertainty behavioral and fMRI data, showing for the first time that practice making decisions under categorical uncertainty in the context of non-equal prior probabilities biases decisions made later when prior probabilities are equal. In simple terms, once you learn a bias it is hard to let it go. The observation of the same tendencies in the perceptual uncertainty data indicates that bias persistency is not unique to categorical uncertainty, but may generalize across decision-making paradigms.

Our manipulation of categorical uncertainty involved ambiguous shapes. It might be asked whether we performed a true test of categorical uncertainty, which would require keeping the shape information constant, but varying the validity of the association between the shape and the correct response. In fact, this description fits our manipulation well. The simplest way to see this is to consider a single categorical shape with curvature in the intermediate (ambiguous) range – for example, the shape with average (13%) curvature. A subject’s experience with this shape is equivalent to the true test of categorical uncertainty. At 50/50 this particular shape is associated with complete uncertainty, while at 80/20 there is less uncertainty for this shape. A similar relationship between the prior condition and the uncertainty level holds for every shape in the intermediate range. Shapes on the extreme ends of the distribution are not ambiguous and therefore are associated with no uncertainty, but this attribute is common to both the categorical and the perceptual uncertainty conditions.

The increased visual cortical activity seen with prior knowledge during perceptual (but not categorical) uncertainty is reminiscent of the increased visual cortical activity seen with top-down, goal-directed, endogenous attention. Conceivably, similar to the effects of directing attention to noisy stimuli (Lu and Dosher, 1998), an adaptive modulation could enhance stimulus attributes indicated by the prior knowledge and/or decrease contraindicated stimulus attributes. Future experiments could explore this issue by systematically investigating the effects of attention on classifying targets during perceptual vs. categorical uncertainty.

## Conflict of Interest Statement

The authors declare that the research was conducted in the absence of any commercial or financial relationships that could be construed as a potential conflict of interest.
